# RNA secondary structure factorization in prime tangles

**DOI:** 10.1186/s12859-022-04879-5

**Published:** 2022-08-18

**Authors:** Daniele Marchei, Emanuela Merelli

**Affiliations:** University of Camerino, Via Madonna delle Carceri 9, 62032 Camerino, Italy

**Keywords:** Brauer monoid, RNA folding, RNA pseudoknots characterization

## Abstract

**Background:**

Due to its key role in various biological processes, RNA secondary structures have always been the focus of in-depth analyses, with great efforts from mathematicians and biologists, to find a suitable abstract representation for modelling its functional and structural properties. One contribution is due to Kauffman and Magarshak, who modelled RNA secondary structures as mathematical objects *constructed* in link theory: *tangles of the Brauer Monoid*. In this paper, we extend the tangle-based model with its minimal prime factorization, useful to analyze patterns that characterize the RNA secondary structure.

**Results:**

By leveraging the mapping between RNA and tangles, we prove that the prime factorizations of tangle-based models share some patterns with RNA folding’s features. We analyze the *E. coli* tRNA and provide some visual examples of interesting patterns.

**Conclusions:**

We formulate an open question on the nature of the class of equivalent factorizations and discuss some research directions in this regard. We also propose some practical applications of the tangle-based method to RNA classification and folding prediction as a useful tool for learning algorithms, even though the full factorization is not known.

## Background

### RNA

**Fig. 1:**
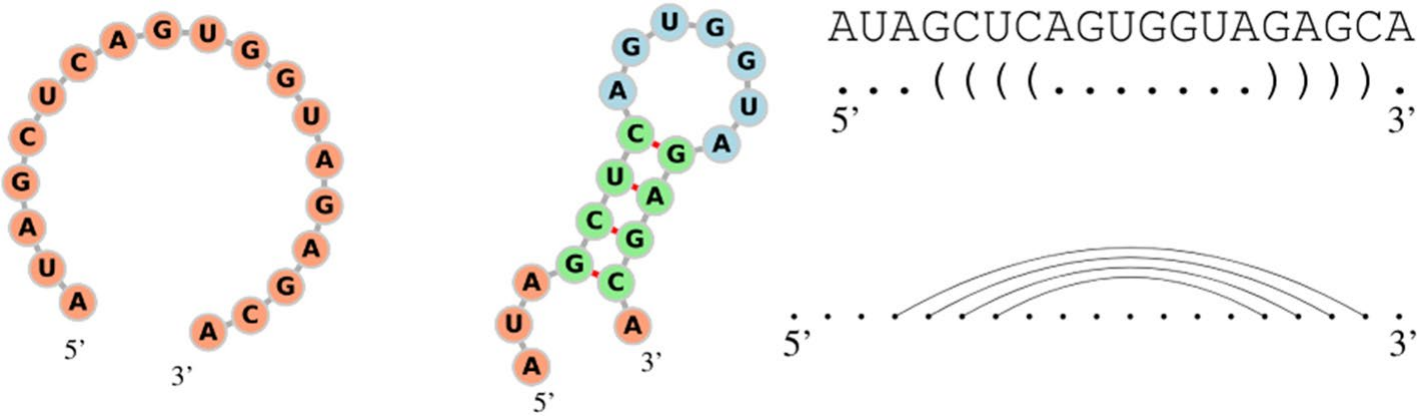
RNA structures, dot-bracket notation and flattened diagram. Example of a RNA found in *Mus musculus* (house mouse) [18]. Its primary structure is on the left and the secondary structure is on the right, along with its dot-bracket representation and flattened diagram. Image generated using FORNA [9]

In biological cells, RNA is a molecule that regulates a huge variety of functions. It consists of a long chain of smaller molecules, called nucleotides, bonded sequentially (Adenine (A), Guanine (G), Cytosine (C), and Uracil (U)), known as the *primary structure*; the first nucleotide of the chain is usually referred as 5’ and the last one as 3’. A *secondary structure* appears when the RNA molecule folds onto itself creating additional *weaker* bonds, called Watson-Crick pairs (A-U, C-G) and Wobble pairs (G-U). Figure 1 shows a primary and secondary structure along with its *dot-bracket notation*, a string in which a pair of matching brackets correspond to a weak bond in the secondary structure and dots unpaired nucleotides. The dot-bracket string can also be represented by a *flattened diagram*, that is a set of points displayed horizontally (representing the nucleotides) joined by an arc in the upper half part of the diagram (representing the pairs). Since every arc has to connect two dots, every flattened diagram has *N* arcs and 2*N* paired dots.

**Fig. 2:**
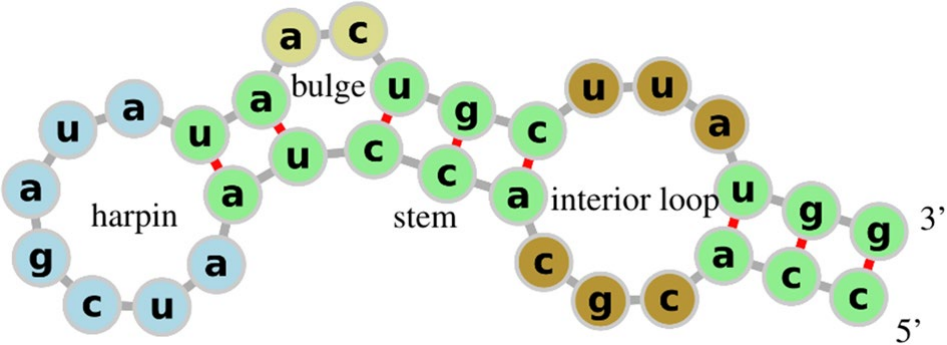
Patterns emerging from a secondary structure. Example of various patterns that can emerge from a secondary structure. Blue nucleotides are part of a hairpin, green ones are part of stems, yellow nucleotides are part of a bulge, brown ones are part of an interior loop

**Fig. 3:**
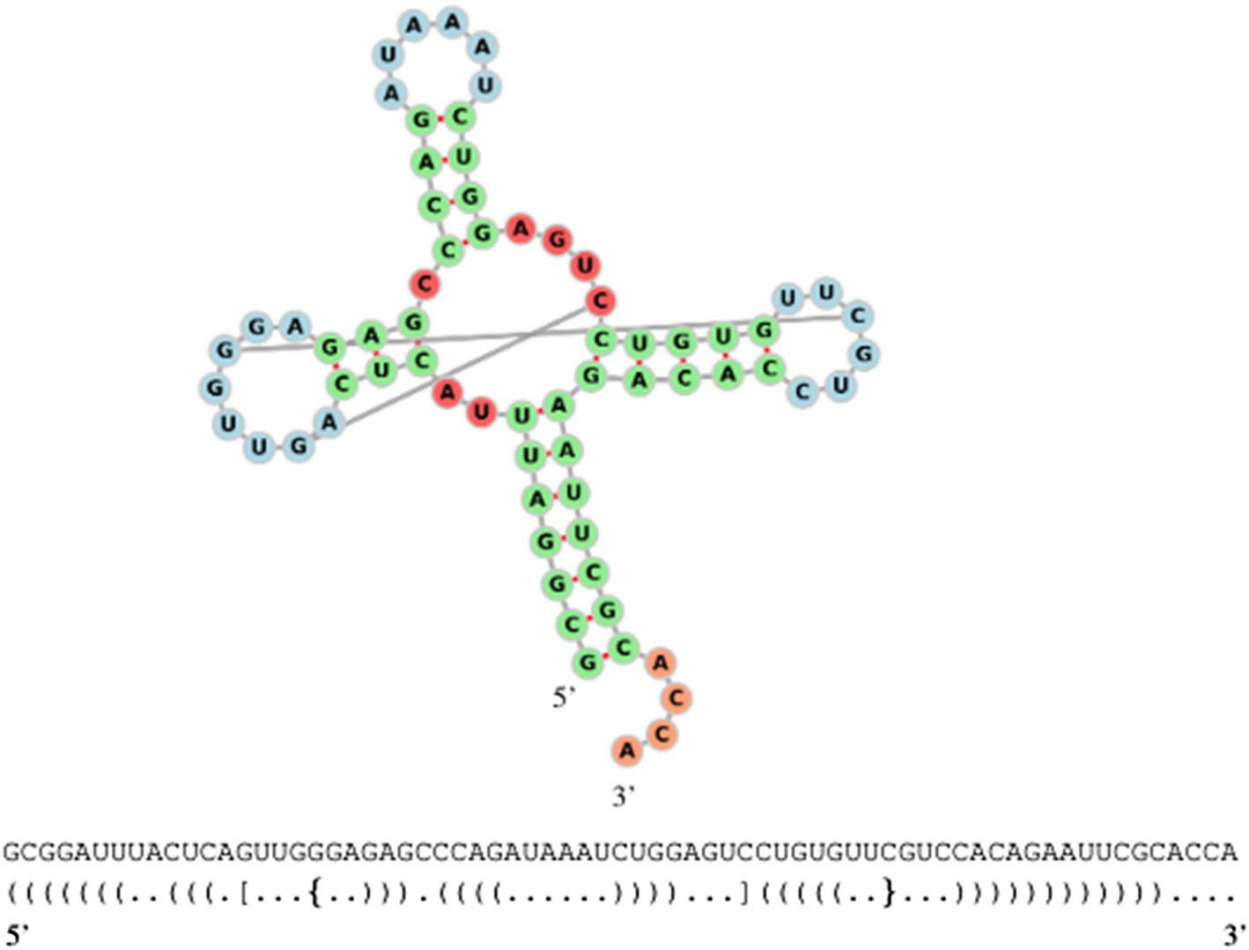
A pseudoknotted tRNA. Secondary structure of the *yeast phenylalanine tRNA* along with its dot-bracket representation [1]. The folding forms a pseudoknot because of the G-C pair at positions 18–50 and pair G-C at position 14–42. There are three multiloops (coloured in red) at the base of the three stems with hairpins

Depending on the bonds present in the secondary structure, different types of brackets may be needed to avoid ambiguity. The folding process gives rise to some interesting structural features (loops) that can be categorized as *hairpins*, *bulges*, *stems*, *interior loops* (see Fig. 2), and *multiloops* (see Fig. 3).

It is often the case that RNA secondary structures form a *pseudoknot*, where an unbonded nucleotide is bonded with another nucleotide in a different loop of the RNA molecule (Fig. 3). Predicting the optimal structure with pseudoknots during the folding process, also known as the *RNA folding problem*, often requires a prohibitive amount of time. Although great efforts were put to solve this problem, both from an algebraic [2, 19, 20, 22] and a machine learning perspective [25], there is still room for improvements.

Due to its pivotal role in biological processes, the study of RNA secondary structures is of great importance. The process of protein production is the result of the interaction of three types of RNA: *transfer RNA*, *ribosomal RNA*, and *messenger RNA*. Viruses have evolved to inject their genome (in the form of RNA) into the host cells in order to replicate themselves. Moreover, it is still in the debate that the self-replicating capabilities of RNA may have given the basis for early life on Earth even before DNA appeared (*RNA World Hypothesis* [11, 14]).

This work proposes a different way to investigate RNA folding with an algebraic structure during the process of optimization, exploiting its decomposition in prime factors.

### Brauer monoid

A monoid is an algebraic structure made by a set of elements and an associative binary operator equipped with an identity element.

**Fig. 4:**
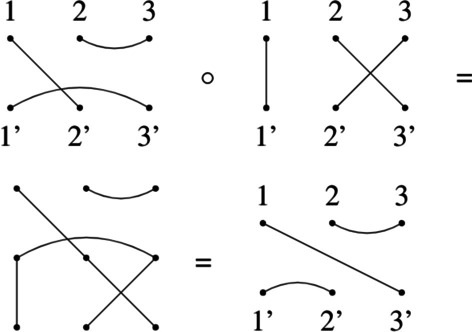
Examples of tangle composition. Composition of two tangles in ${\mathcal {B}}_{3}$. The first tangle is put on top of the second one, then the resulting edges are redrawn to minimize intersections

**Fig. 5:**
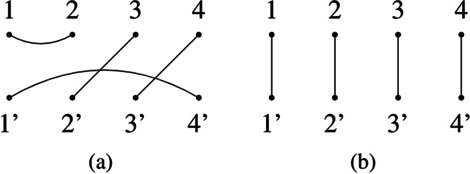
Examples of tangles. **a** A graphical representation of a tangle in ${\mathcal {B}}_{4}$. Its invariant is $1:2,3:2',4:3',1':4'$. **b** $I_4 = 1:1', 2:2',3:3',4:4'$, the identity tangle for ${\mathcal {B}}_{4}$

Given a natural *N* and a set of 2*N* dots in $[N] \cup [N]'$, where $[N] = \{1,2,\ldots ,N\}$ and $[N]' = \{1',2',...,N'\}$, a *tangle* is a set of *N* pairs (called edges) of distinct dots, such that no dot occurs in more than one edge. Tangles are represented graphically by drawing two rows of *N* dots labelled with [*N*] if they are on the top and labelled with $[N]'$ if they are on the bottom. All edges are represented by lines connecting pairs of dots. The edge enumeration of a tangle is called *invariant* and we will represent it by separating edges by commas and pair of dots by colons (see Fig. 5). We can compose two tangles by identifying the bottom row of the first with the top row of the second one and then redraw the edges accordingly (see Fig. 4). The set of all tangles on 2*N* points under the composition operator $\circ$ is called the *Brauer Monoid* ${\mathcal {B}}_{N}$ [3].

Edges in the form $e = a:b'$ are called transversals, and in the cases when $a > b'$, $a < b'$ or $a = b'$ we call them positive, negative, and zero transversal respectively. Edges in the form $e = a:b$ or $e = a':b'$ are called upper and lower hooks respectively [6]. The size of an edge $e = a:b$, with *a* and *b* arbitrary dots, is defined as $|e| = |a-b|$.

${\mathcal {B}}_{N}$ is closed under composition and its *identity* is $I_N = 1:1', 2:2',..., N:N'$.

**Fig. 6:**
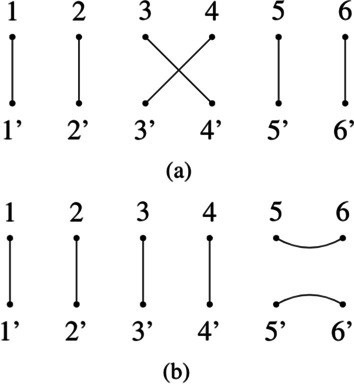
Examples of prime tangles. Two prime tangles in ${\mathcal {B}}_{6}$. **a** A ${\mathcal {T}}$-*prime* $T_3$ and **b** a ${\mathcal {U}}$-*prime* $U_5$

A tangle *P* is called *prime* if it can only be written in the form $P = I_N \circ P = P \circ I_N$. There are two types of primes tangles (Fig. 6):

- $$T_i = 1:1',2:2',...,i:i'+1,i+1:i',...N:N'$$
- $$U_i = 1:1',2:2',...,i:i+1,...,i':i'+1,...N:N'$$

called respectively ${\mathcal {T}}$-*prime* and ${\mathcal {U}}$-*prime*. ${\mathcal {B}}_{N}$ contains exactly $N-1$ ${\mathcal {T}}$-*prime* and $N-1$ ${\mathcal {U}}$-*prime*.

Note that crossings in a tangle are only introduced by ${\mathcal {T}}$-*primes*. ${\mathcal {T}}$-*primes* and ${\mathcal {U}}$-*primes* are the generators for all tangles in ${\mathcal {B}}_{N}$ under composition, this means that we can reduce any tangle to a prime factorization. It is useful to note here that factorization in the Brauer Monoid is not unique.

A factor list ${\mathbf {F}}$ for a tangle *X* is a list of prime tangles in the form $P_{x_1} \circ P_{x_2} \circ \cdots \circ P_{x_i}$ such that their composition gives back *X*. The length of a factor list ${\mathbf {F}}$ is indicated by $|{\mathbf {F}}|$. The factor list ${\mathbf {F}}$ of the identity tangle $I_N$ is the empty list, whose size is $|{\mathbf {F}}| = 0$.

For each tangle $X \in {\mathcal {B}}_{N}$, we call the *factorization problem* the task of finding the factor list of minimal length.

## Methods

The first attempt to draw a connection between RNA secondary structures and tangles in the Brauer Monoid was due to Kauffman and Magarshak [12]. Their intuition was that the number of parenthesis in RNA dot-bracket representation and the number of dots in a tangle is always even, and each open parenthesis must correspond to a closed parenthesis somewhere in the string, corresponding with the existence of an edge in a tangle. Therefore, they provided the following procedure for converting an RNA secondary structure to a tangle:

1. flatten the secondary structure in a single long chain (equivalent to the dot-bracket notation);
2. discard the unpaired nucleotides, there are now 2*N* nucleotides and *N* pairs;
3. abbreviate stacked arcs to a single arc. We will call this reduced diagram *shape* [10, 21];
4. rotate the second half of the shape diagram above the first;
5. enumerate the nucleotides in the top row with numbers in [*N*] and nucleotides in the bottom row with numbers in $[N]'$.

As Giegerich et al. pointed out, the study of the shape of an RNA secondary structure lifts the user from the burden of paying attention to changes that do not affect the overall desired structure, which means that we do not lose information because we are doing a static analysis [10]. In this context, the procedure described above gives us the opportunity to study the shape of RNA secondary structures in terms of tangles and generators for these tangles. For this purpose, we wrote an algorithm capable of finding the minimal amount of prime compositions for any given tangle [16]. We classify tangles in the following way:

$${\mathcal {T}}$$*-tangle*:
a tangle $X = T_a \circ T_b \circ \cdots \circ T_i$ (all edges of *X* are transversal);
$${\mathcal {U}}$$*-tangle*:
a tangle $X = X' \circ U_i$ (*X* has a lower hook *h* of size $|h| = 1$);
$${\mathcal {T}}{\mathcal {L}}$$*-tangle*:
a ${\mathcal {U}}$*-tangle* with the extra condition of having only ${\mathcal {U}}$-*primes* as factors (no edge in *X* intersect with another edge. ${\mathcal {T}}{\mathcal {L}}$ stands for *Temperley-Lieb*, those who first described them [23]);
$${\mathcal {H}}$$*-tangle*:
all the other tangles (${\mathcal {H}}$ stands for *big hook* because they will always have a lower hook *h* of size $|h| > 1$.)

**Fig. 7:**
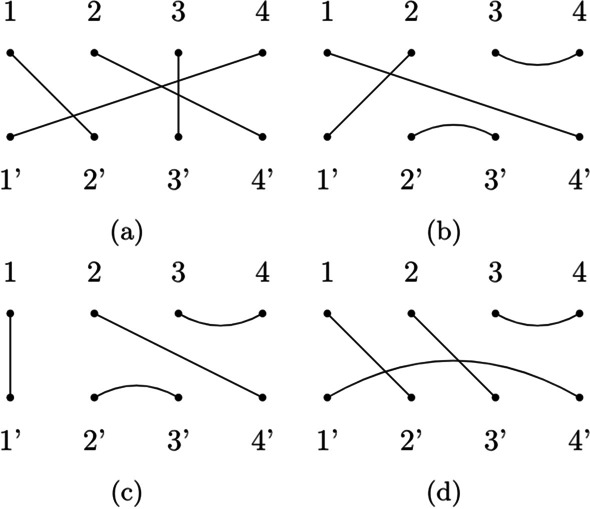
Types of tangles. A display of our tangle classification criteria. **a** A ${\mathcal {T}}$*-tangle*, **b** a ${\mathcal {U}}$*-tangle*, **c** a ${\mathcal {T}}{\mathcal {L}}$*-tangle*, **d** a ${\mathcal {H}}$*-tangle*

For a visual example see Fig. 7. For each class of tangles, we provide an algorithm for calculating its factorization.

### Factoring ${\mathcal {T}}$-*tangles*

The set of ${\mathcal {T}}$-*tangles* on 2*N* dots is actually isomorphic to the symmetric group $S_N$, therefore we can represent any ${\mathcal {T}}$*-tangle* *X* as a permutation in the form

1 $$\begin{aligned} \begin{pmatrix} 1 &{} 2 &{} ... &{} 2N \\ x_1' &{} x_2' &{} ... &{} x_{2N}' \end{pmatrix} \end{aligned}$$

and we can find an optimal factorization by sorting the bottom row of *X*. Since every ${\mathcal {T}}$-*prime* is equivalent to an adjacent swap, we are limited to ${\mathcal {O}}(N^2)$ algorithms, like BubbleSort.
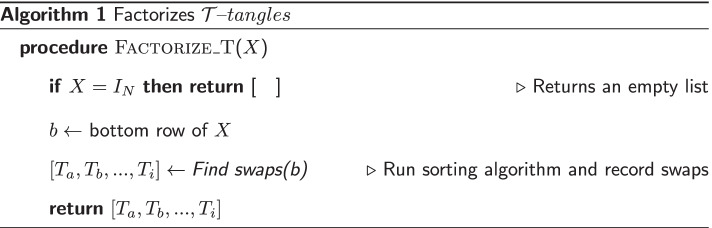


### Factoring ${\mathcal {T}}{\mathcal {L}}$*-tangles*

Ernst et al. defined a factorization algorithm that constructs a minimal factor list given an input ${\mathcal {T}}{\mathcal {L}}$-*tangle* [7]. Their algorithm works by subdividing the tangle to factorize in vertical columns and then enumerating all regions of odd depth (called 1-*regions*) that this subdivision generates. Each region will correspond to a ${\mathcal {U}}$-*prime*, and if two regions $R_1$ and $R_2$ are diagonally adjacent, with $R_1$ having a lower depth than $R_2$, then they write $R_1 \rightarrow R_2$, therefore constructing a Directed Acyclic Graph (DAG) of regions. By reading this graph left to right and from top to bottom, they obtain a minimal factor list. Our implementation of their algorithm takes quadratic time. For a more detailed explanation, the reader can refer to the original paper.
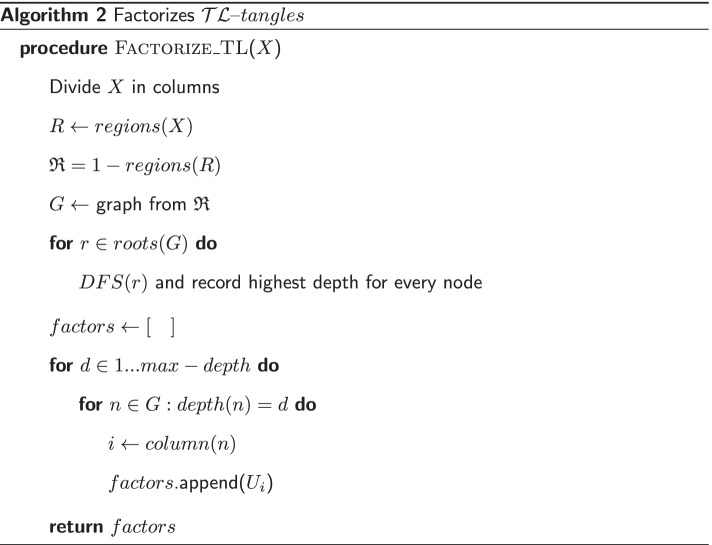


### Factoring ${\mathcal {U}}$*-tangles*

Recall that a ${\mathcal {U}}$*-tangle* is a tangle in the form $X = X' \circ U_i$, we would like to find $X'$ by removing $U_i$ from *X*. To do this, we will merge the lower hook $h = i':i'+1$ with another edge in the tangle.

We say that we merge a lower hook $h=i':i'+1$ and an edge $e=e_1:e_2$ by removing them from *X* and adding edges *a* and *b* such that if *e* is a hook or a negative transversal, then $a=e_1:i'$ and $b=i'+1:e_2$ and if *e* is a positive transversal, then $a=e_1:i'+1$ and $b = e_2:i'$.

Since the number of crossings in a tangle corresponds to the number of ${\mathcal {T}}$-*primes* in its factor list, we would like this merging process to maintain the crossing number constant, in this way we are sure to not include any more ${\mathcal {T}}$-*primes* in the non-optimal factor list we are calculating.

#### **Heuristic 1**

*Let* $X = X' \circ U_i$ *be a* ${\mathcal {U}}$-*tangle* *with* *c* *number of crossings and with a lower hook* $h = i':i'+1$. *Let* $I = \{i:i', i+1:i'+1\}$. *For all edges* $e \ne h$ *calculate* *inter*(*e*) *to be the number of intersections* *e* *has with edges in* *I*. Let $S = \{e : e \in X, inter(e) = 2\}$ *be the set of edges that intersect both edges in* *I*, *for each* $e \in S$ *calculate the number of crossings the tangle* $X'$ *would have if we merged* *h* *with* *e* *and pick the tangle whose number of crossings is equal to* *c*. *If more than one edge satisfies this last condition, among them, pick the edge that has the least amount of intersections in* *X*.

Note that, for the case of edges in *I*, it will happen that some edges in *X* will share a dot with edges in *I*. We count them too as intersecting.

Merging two edges takes constant time, but the calculation of the crossing number takes ${\mathcal {O}}(N^2)$ [24], and since we have to merge *h* with *N* edges in the worst case, the time complexity for this heuristic is ${\mathcal {O}}(N^3)$.

### Factoring ${\mathcal {H}}$-*tangles*

We will extract factors from a ${\mathcal {H}}$*-tangle* *X* by transforming it into a ${\mathcal {U}}$*-tangle*. The idea is to take one of the lower hooks *h* with size $|h| > 1$ and *shrink* it until it becomes of size one. To do this we compose *X* with ${\mathcal {T}}$-*primes* until this condition is met. During the shrinkage process, other edges will inevitably change size. In order to decide *where* we should shrink *h*, we use a heuristic that chooses a location where the size of the other edges increases the least. We apply this heuristic to the smallest lower hook of *X*, in this way there will be no smaller lower hook inside of it.

#### **Heuristic 2**

*Given a* ${\mathcal {H}}$*-tangle* *X*, *let* $h = i':i'+k$ *be the smallest lower hook of* *X* *of size* $k > 1$. *Let* *j* *be the index of the shrinkage location where the size of the other edges increases the least. Shrink the lower hook* *h* *into location* *j* *by composing* *X* *with* ${\mathbf {L}} = T_i \circ T_{i+1} \circ \cdots \circ T_{i+j-1}$ *and* ${\mathbf {R}} = T_{i+k-1} \circ T_{i+k-1} \circ \cdots \circ T_{j+1}$. *This procedure yields a* ${\mathcal {U}}$*-tangle* $X'$ *such that* $X = X' \circ {\mathbf {L}}^{-1} \circ {\mathbf {R}}^{-1}$.

The notation ${\mathbf {F}}^{-1}$ indicates the reverse of a factor list, given ${\mathbf {F}} = P_{x_1} \circ P_{x_2} \circ \cdots \circ P_{x_i}$ then ${\mathbf {F}}^{-1} = P_{x_i} \circ \cdots \circ P_{x_2} \circ P_{x_1}$.

This heuristic is not optimal, but it can be computed in linear time.

**Table 1:** Rules for prime tangles

Rule type	Rule id	Rule				
Delete	R1	$T_i \circ T_i$	=	$I_N$		
	R2	$U_i \circ U_i$	=	$U_i$		
	R3	$T_i \circ U_i$	=	$U_i$		
	R4	$U_i \circ T_i$	=	$U_i$		
	R5	$U_i \circ U_j \circ U_i$	=	$U_i$	$\iff$	$|i-j| = 1$
	R6	$U_i \circ T_j \circ U_i$	=	$U_i$	$\iff$	$|i-j| = 1$
	R7	$T_i \circ U_j \circ U_i$	=	$T_j \circ U_i$	$\iff$	$|i-j| = 1$
	R8	$U_i \circ U_j \circ T_i$	=	$U_i \circ T_j$	$\iff$	$|i-j| = 1$
	R9	$U_i \circ T_j \circ T_i$	=	$U_i \circ U_j$	$\iff$	$|i-j| = 1$
	R10	$T_i \circ T_j \circ U_i$	=	$U_j \circ U_i$	$\iff$	$|i-j| = 1$
Move	R11	$T_i \circ T_j \circ T_i$	=	$T_j \circ T_i \circ T_j$	$\iff$	$|i-j| = 1$
	R12	$T_i \circ U_j \circ T_i$	=	$T_j \circ U_i \circ T_j$	$\iff$	$|i-j| = 1$
	R13	$P_i \circ P_j$	=	$P_j \circ P_i$	$\iff$	$|i-j| > 1$

### Minimal factorization

The heuristics mentioned above do not always yield a minimal factorization, therefore a minimization step is required. It turns out that prime tangles follow a particular set of rules (see Table 1) [13]. We call R1–10 *delete rules* and R11–13 *move rules*. We can use them to minimize a non optimal factor lists by implementing them in a rewriting logic tool (we chose the *Maude System* [5, 15]).

### From RNA to tangle factorization

We will now provide an example of the mapping procedure for deriving, from a RNA secondary structure, a tangle with its prime factors.

**Fig. 8:**
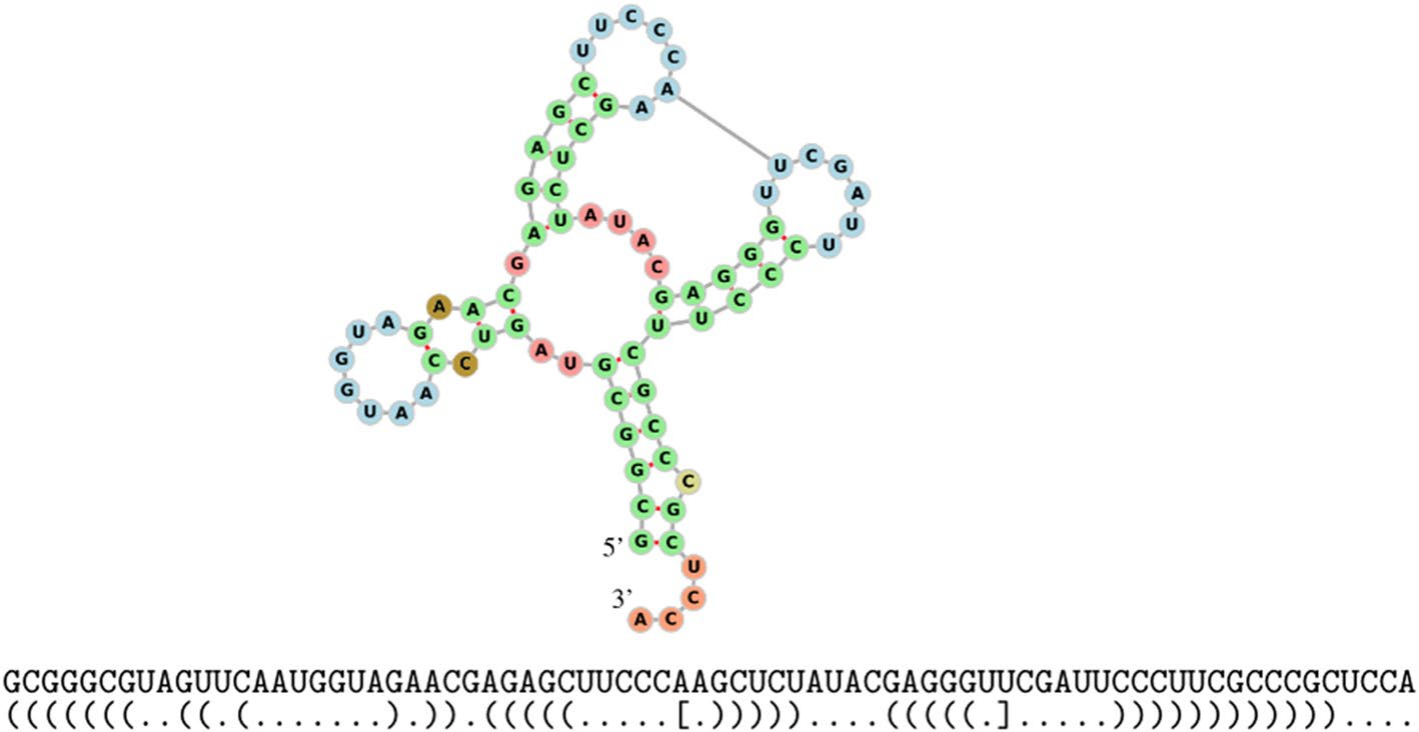
Modified *E. coli* tRNA. Pseudoknotted secondary structure for a modified *E. coli* tRNA along with its dot-bracket representation

**Fig. 9:**
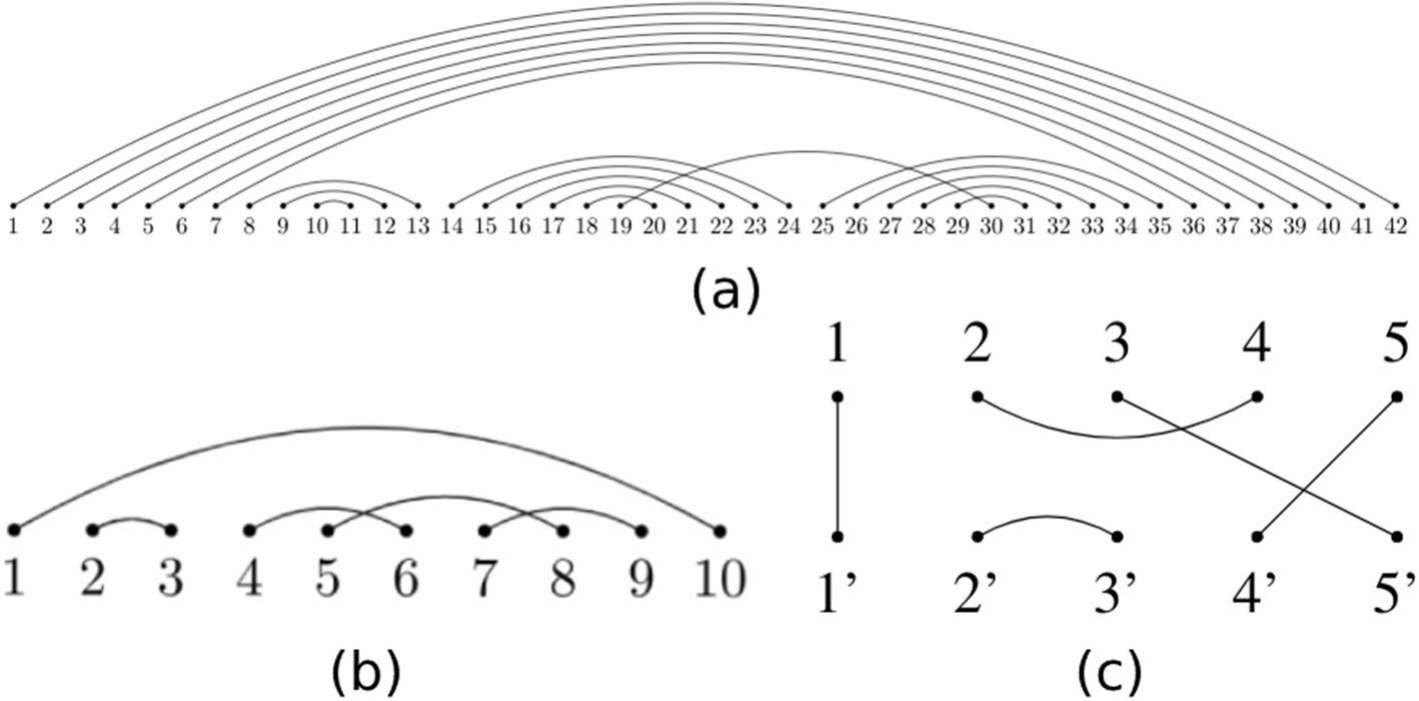
Flattened diagram, shape diagram, and corresponding tangle. **a** The flattened diagram for the modified *E. coli* tRNA. **b** The shape diagram is computed by merging together all parallel edges in the flattened diagram. **c** The corresponding ${\mathcal {U}}$*-tangle* in ${\mathcal {B}}_{5}$

We will start from the modified *E. coli* tRNA in Fig. 8 [8], and apply Kauffman and Magarshak’s mapping to obtain the flattened diagram in Fig. 9a.

**Fig. 10:**
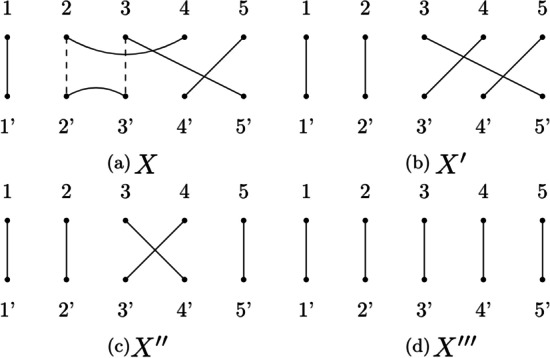
Factorization steps. The steps (from **a** to **d**) that our algorithm takes in order to factorize the tangle (**a**)

This diagram is reduced to obtain a shape diagram (Fig. 9b) that can be folded to get the corresponding tangle (Fig. 9c). We can now factorize it by using the methods discussed previously (Fig. 10).

Figure 10 shows the four steps of the factorization algorithm:

- The algorithm recognizes that *X* is a ${\mathcal {U}}$*-tangle* because there is a lower hook of size 1 ($2':3'$). Therefore it can be rewritten as $X = X' \circ U_2$. The algorithm applies Heuristic 1 that determines that the upper hook 2 : 4 in the only one intersecting the two imaginary edges (the two vertical dotted lines) twice. Therefore these two edges are merged and we obtain the tangle $X'$. The prime $U_2$ is yielded and the algorithm moves to the next step.
- The rewritten tangle $X'$ is a ${\mathcal {T}}$*-tangle*. The algorithm applies BubbleSort that firstly extracts ${\mathcal {T}}_4$, thus shrinking the edge $3:5'$ to $3:4'$ and obtaining $X''$.
- The BubbleSort applies one more swap, which corresponds to a $T_3$ and delivers $X'''$
- The algorithm has now reached the identity tangle ($X'''$) and the first part of the factorization process has terminated.

**Fig. 11:**
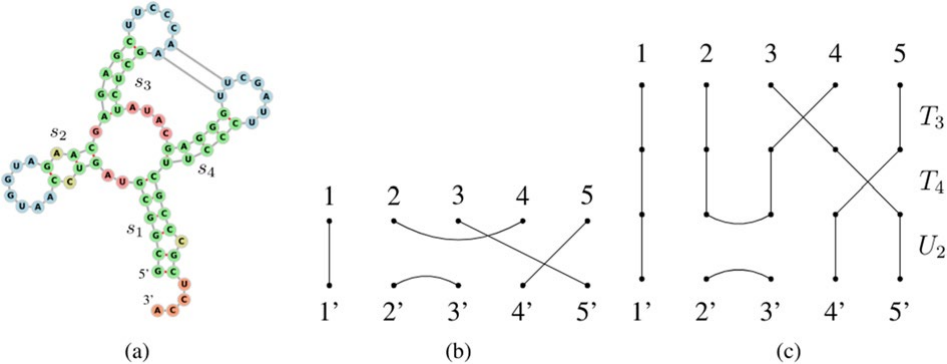
Example of a tRNA with its corresponding tangle and factorization. **a** A modified *E. coli* tRNA. **b** The correspondent abbreviated tangle, with minimal factorization $T_3 \circ T_4 \circ U_2$. **c** The three factors composed. This makes it easier to visualize the path that each edge takes

Thus the yielded factorization is $T_3 \circ T_4 \circ U_2$. Now the algorithm moves to the rewriting logic step, whose aim is to ensure that this is the minimal factorization and, if it is not, to find a better one. Since there is no move rule that can lead to the application of a delete rule, the algorithm concludes that this factor list is minimal (Fig. 11c).

An online interactive demo that calculates these steps automatically is available [17].

### Examples

**Fig. 12:**
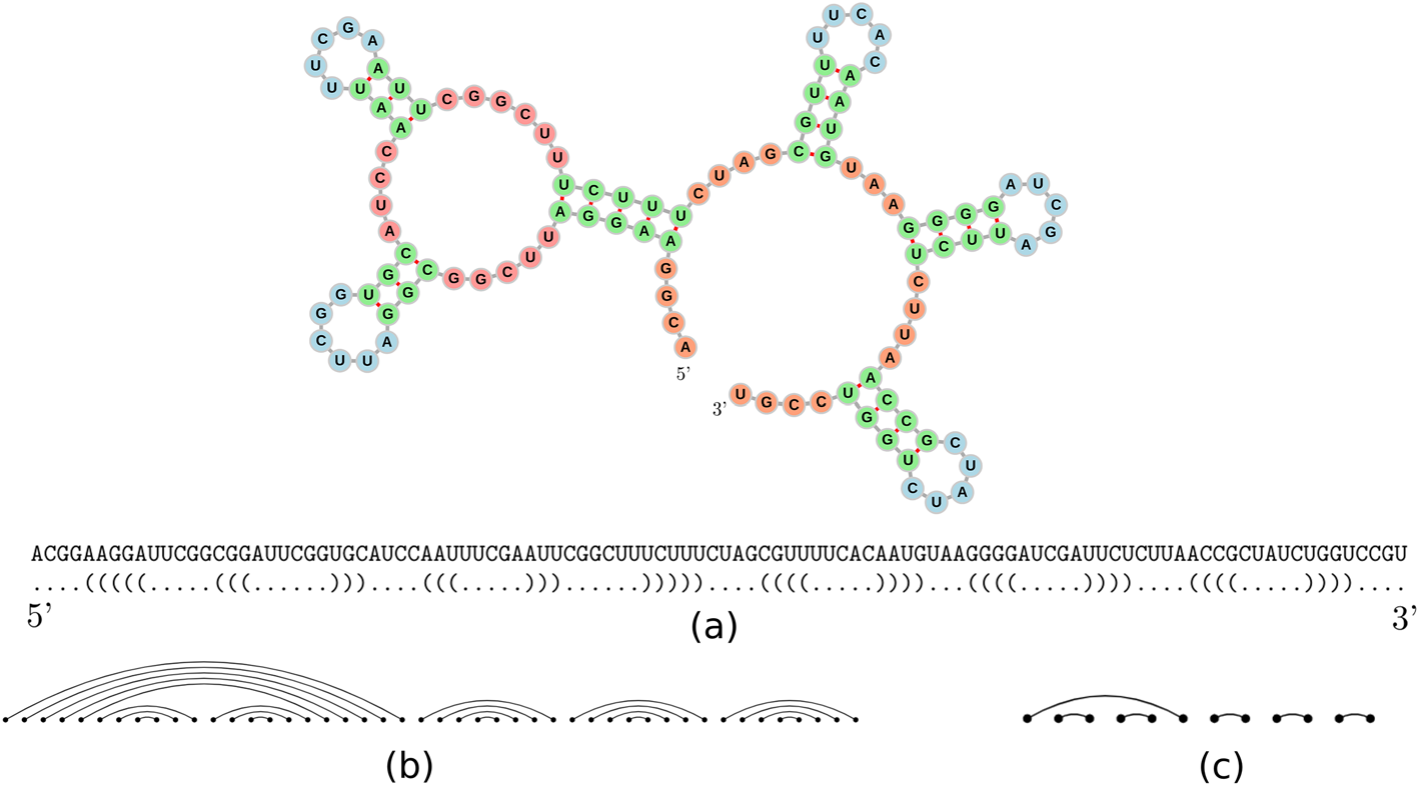
Example 1: RNA. **a** A pseudoknot free RNA secondary structure along with its primary structure and dot-bracket representation. **b** The flattened diagram (unpaired nucleotides are not drawn due to space constraints). **c** The shape diagram obtained by collapsing parallel edges onto a single one

**Fig. 13:**
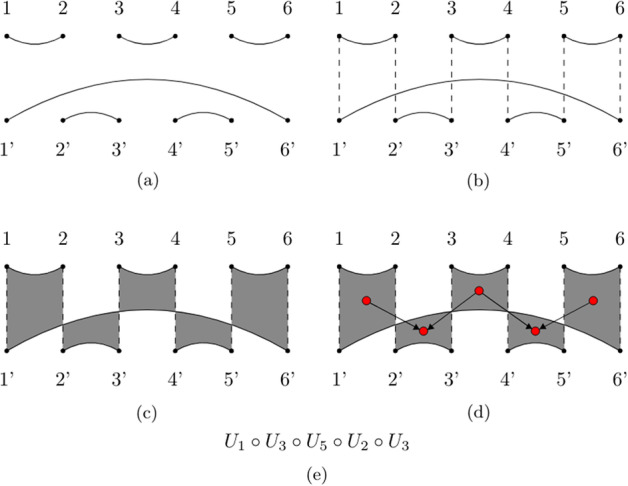
Example 1: Factorization. **a** The tangle obtained from the shape diagram. **b** The tangle divided into five columns. **c** The regions of odd depth are colored in gray. **d** The DAG obtained by Ernst’s algorithm. **e** The minimal factorization for the initial tangle

*RNA without pseudoknots* Figure 12a is an example of a RNA molecule that does not have any pseudoknots, therefore its corresponding tangle will not have any crossings. This implies that it will be mapped to a ${\mathcal {T}}{\mathcal {L}}$*-tangle*, which we know can be factorized using Ernst’s algorithm. To obtain the corresponding tangle we apply Kauffman and Magarshak’s mapping. We take its secondary structure (represented as a flattened diagram in Fig. 12b) and reduce it to a shape diagram (Fig. 12c). The shape diagram can now be folded in half to obtain the tangle in Fig. 13a. We then apply Ernst’s algorithm by dividing it into five columns (Fig. 13b), i.e. by drawing imaginary edges that connect each upper dot to its corresponding bottom dot, and selecting for each of them the regions of odd depth (Fig. 13c). We then build the DAG by connecting two regions $R_1$ and $R_2$ if they are diagonally adjacent and $R_1$ is above $R_2$ (Fig. 13d). To each node will now correspond a region, and each edge will indicate when two regions are diagonally adjacent. We then read the graph nodes from top to bottom and from left to right. If a node is in column *i*, then we will write in output the prime tangle $U_i$ (Fig. 13e).

*RNA with pseudoknots*

**Fig. 14:**
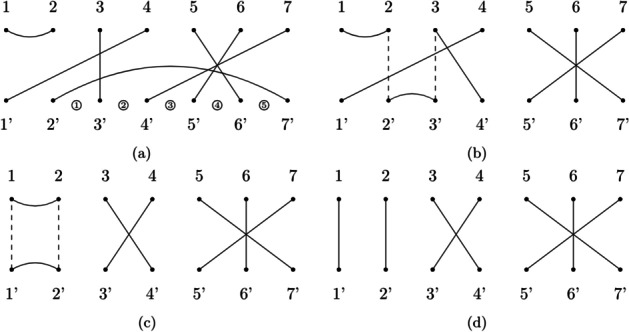
Example 2. **a** The ${\mathcal {H}}$*-tangle* to be factorized. Circled numbers index all possible shrinkage locations for the lower hook $2':7'$. **b** Apply the Heuristic for ${\mathcal {U}}$-*tangles*. The dashed lines indicate the edges in the set $I = \{2:2', 3:3'\}$. **c** Apply the Heuristic for ${\mathcal {U}}- tangles$ again. **d** The resulting ${\mathcal {T}}$*-tangle*, it can be factorized optimally by using Algorithm 1

Suppose to have a complex RNA secondary structure that yields the tangle in Fig. 14a. Since it is a ${\mathcal {H}}- tangle$, for this example our algorithm applies Heuristic 2 on the smallest lower hook (in this case there is only one, namely $2':7'$). To choose where we should shrink this lower hook, the algorithm calculates which shrinkage location increases the size of the other edges the least (Table 2).

**Table 2:** This table calculates, for each edge inside lower hook $2':7'$, how much it would increase (or decrease) in size if the algorithm shrunk lower hook $2':7'$ into shrinkage locations from 1 to 5

Shrinkage location	3:3′	7:4′	6:5′	5:6′	Sum	Factors
1	+1	-1	-1	+1	0	$T_6 \circ T_5 \circ T_4 \circ T_3$
2	+1	-1	-1	+1	0	$T_2 \circ T_6 \circ T_5 \circ T_4$
3	+1	+1	-1	+1	2	$T_2 \circ T_3 \circ T_6 \circ T_5$
4	+1	+1	+1	+1	4	$T_2 \circ T_3 \circ T_4 \circ T_6$
5	+1	+1	+1	-1	2	$T_2 \circ T_3 \circ T_4 \circ T_5$

The best shrinkage location is selected among those who have the minimal sum of these sizes (1 and 2 in this case). The rightmost column indicates which set of prime factors, when composed with the initial tangle, shrink the $2':7'$ in the selected location

Since in this case the heuristic found two best locations, 1 and 2, it randomly chooses location number 1. Therefore $2':7'$ will be shrunk to a lower hook $2':3'$ and the factorization yielded so far is $T_3 \circ T_4 \circ T_5 \circ T_6$, the reverse of the factorization for this location (we record the reverse because if during factorization we need to shrink the lower hook, during composition we need to *expand* it). The algorithm now tries to factorize the tangle returned from the last step (Fig. 14b). Since it is a ${\mathcal {U}}$*-tangle*, the algorithm will apply Heuristic 1. It will select the lower hook $2':3'$ and check which edges intersect with the imaginary edges in the set $I = \{2:2', 3:3'\}$. The only edge intersecting both is $4:1'$, therefore $2':3'$ and $4:1'$ are merged together. This step returns the tangle in Fig. 14c and yields the prime factor $U_2$. Since the tangle in Fig. 14c is still a ${\mathcal {U}}$*-tangle*, the same step is applied again, returning the ${\mathcal {T}}$*-tangle* in Fig. 14d and yielding the prime factor $U_1$. This last tangle can be factorized optimally by applying Algorithm 1, which yields the factorization $T_3 \circ T_5 \circ T_6 \circ T_5$ by performing the following steps:

$$\begin{aligned} \begin{array}{ll} 1'\ 2'\ \underline{4'\ 3'}\ 7'\ 6'\ 5'\ &{} T_3\\ 1'\ 2'\ 3'\ 4'\ \underline{7'\ 6'}\ 5'\ &{} T_5\\ 1'\ 2'\ 3'\ 4'\ 6'\ \underline{7'\ 5'}\ &{} T_6\\ 1'\ 2'\ 3'\ 4'\ \underline{6'\ 5'}\ 7'\ &{} T_5\\ 1'\ 2'\ 3'\ 4'\ 5'\ 6'\ 7'\ &{} {\text {STOP}}\\ \end{array} \end{aligned}$$

This last step returns the identity tangle, therefore the algorithm stops and yields the factorization $T_3 \circ T_5 \circ T_6 \circ T_5 \circ U_1 \circ U_2 \circ T_3 \circ T_4 \circ T_5 \circ T_6$. This factorization is minimal therefore the reduction step is not necessary.

*Reduction of a non-minimal factorization* Suppose the following non-minimal factorization term is given: $T_2 \circ U_1 \circ U_1 \circ U_2 \circ U_3 \circ U_1 \circ U_2 \circ T_4$. The rewriting logic step minimizes the term by performing the following rewrites using the rules presented in Table 1.

$$\begin{aligned} \begin{array}{lcl} T_2 \circ \underline{U_1 \circ U_1} \circ U_2 \circ U_3 \circ U_1 \circ U_2 \circ T_4 &{} {\text {R2}} &{} U_i \circ U_i = U_i\\ T_2 \circ U_1 \circ U_2 \circ \underline{U_3 \circ U_1} \circ U_2 \circ T_4 &{} {\text {R13}} &{} P_i \circ P_j = P_j \circ P_i \iff |i-j| = 1 \\ T_2 \circ \underline{U_1 \circ U_2 \circ U_1} \circ U_3 \circ U_2 \circ T_4 &{} {\text {R5}} &{} U_i \circ U_j \circ U_i = U_i \iff |i-j| = 1 \\ T_2 \circ U_1 \circ U_3 \circ U_2 \circ T_4 &{} {\text {STOP}} &{} {-}\\ \end{array} \end{aligned}$$

In the last step there are no delete rules applicable and no move rules that eventually lead do a delete. Therefore this factor list is minimal.

## Results

The resulting tangle is invariant to *synonymous* mutations, which are mutations that do not change the secondary structure. This is due to the fact that we discard unpaired nucleotides and abbreviate stacked arcs, allowing multiple secondary structures to map to the same factorization. This also allows researchers to move their attention to patterns in the factorizations of their desired shapes. A less obvious result (already observed by Kauffman and Magarshak) is that every secondary structure without pseudoknots maps to a ${\mathcal {T}}{\mathcal {L}}$*-tangle*. The intuition behind this result is that the number of valid ways we can arrange 2*N* open and closed parenthesis of a single type is the Catalan number

2 $$\begin{aligned} C_N = \frac{1}{N+1}{2N \atopwithdelims ()N} \end{aligned}$$

which is exactly the number of tangles with non-crossing edges in ${\mathcal {B}}_{N}$ [4, 23]. This also implies that every pseudoknotted secondary structure corresponds to a tangle with at least one crossing, and thus at least one ${\mathcal {T}}$-*prime* as a factor.

Let us show some other properties using the example we provided in the previous section (Fig. 11). In the corresponding tangle, only stems and pseudoknots are visible and they are encoded in the factorization. Starting from stem $s_1$, six pairs are identified with the unique vertical edge, which does not have corresponding factors. Its presence, however, causes the indexes of the prime tangles to be shifted by one (Proposition 1). The three pairs of the stem $s_2$ correspond to the $2':3'$ arc generated by the factor $U_2$. The stem $s_3$, corresponding to the edge $5:4'$, is generated by $T_4$. This is because its two endpoints were situated in the first and second half of the flattened secondary structure, causing it to be represented as a diagonal edge. The stem $s_4$, identified with the edge 2 : 4, is generated by $T_3 \circ U_2$ (note that $T_4$ and $U_2$ can commute, see “Discussion” section). Lastly, the pseudoknots are identified with edge $3:5'$ generated by $T_3$ and $T_4$, which are the factors in common with the edges that it crosses, 2 : 4 and $5:4'$ (Proposition 3).

We will give a mathematical foundation for these empirical results. Given a section *s* of an RNA secondary structure, stem or pseudoknot, we write $edge(s) = (i,j)$ to denote its corresponding edge in the RNA shape (or tangle) beginning in position *i* and ending in position *j* (with $i < j$). Given a tangle *X* and an edge $e \in X$, we will write *gen*(*e*) to indicate the factors that generate it.

### **Proposition 1**

*If an RNA secondary structure has a stem* *s* *with* $edge(s) = (1,2N)$, *then the index of every factor of the corresponding tangle* $X \in {\mathcal {B}}_{N}$ *will always be greater or equal to two. The converse is also true.*

### *Proof*

Assume that an RNA shape has an edge $e = (1,2N)$. Let *X* be the corresponding tangle, then $1:1' \in X$ and therefore there is no prime $T_1$ or $U_1$ in the factorization of *X*. The backward argument is also valid. $\square$

### **Proposition 2**

*Let* *s* *be a stem of an RNA secondary structure and let* *p* *be a pseudoknot starting inside the hairpin of* *s* *and ending outside of it. Then* *edge*(*s*) *will cross* *edge*(*p*).

### *Proof*

We can abstract *edge*(*s*) to be a 2-dimensional closed curve ${\mathcal {S}} \subset {\mathbb {R}}^2$ by closing its two ends with a horizontal line. We then have that *edge*(*p*) starts inside of ${\mathcal {S}}$ and ends outside of it. By the Jordan Curve Theorem on ${\mathbb {R}}^2$ we know that *edge*(*p*) must cross ${\mathcal {S}}$, and since we assume that in the shape diagram all edges are situated in the upper portion of the diagram we know that *edge*(*p*) must cross *edge*(*s*). $\square$

### **Proposition 3**

*Let* *X* *be a tangle with* $e_1, e_2 \in X$ *and let* $G = gen(e_1) \cap gen(e_2)$. *If* $e_1$ *and* $e_2$ *cross, then there exists* $T_i \in G$ *for some* *i*.

### *Proof*

Since $e_1$ and $e_2$ cross, they must share a prime tangle *P* that generates their crossing. But since every intersection is generated by a ${\mathcal {T}}$-*prime*, *P* must be a ${\mathcal {T}}$-*prime*. This implies that a ${\mathcal {T}}$-*prime* generates both $e_1$ and $e_2$. $\square$

## Discussion

The existence of equivalent factorizations leads us to reason about an open question:

### Open Question

What is the biological interpretation of *commutative factors* and, in general, of *equivalent factorizations*?

We hypothesise two separate research directions, regarding:

- equivalent factorizations up to commutativity (R13)
- equivalent factorizations up to R11 and R12

The reason for this distinction is that R13 does not really impose a challenge during factorization, recall that R13 is defined as:

$$\begin{aligned} {\text {R13}}.\quad P_i \circ P_j = P_j \circ P_i \iff |i - j| > 1 \end{aligned}$$

The number of prime factors $P_i$ and $P_j$ remains unchanged, whereas in R11 and R12:

$$\begin{aligned} {\text {R11}}.\quad T_i \circ T_j \circ T_i = T_j \circ T_i \circ T_j \iff |i - j| = 1 \\ {\text {R12}}.\quad T_i \circ U_j \circ T_i = T_j \circ U_i \circ T_j \iff |i - j| = 1 \end{aligned}$$

The number of $T_i$s is two on the left side and one on the right for R11, and for R12, the left side and the right side do not even share a common factor. Since the factorization yielded by R11 and R12 is fundamentally different, we think that they have a different biological interpretation than R13.

**Fig. 15:**
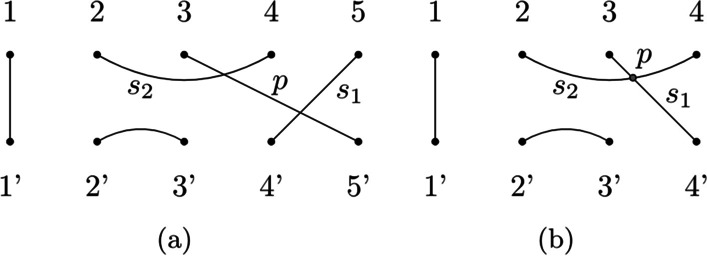
Two different mappings. Two mapping in which pseudoknots are treated differently. $s_1$ and $s_2$ are two stems and *p* is a pseudoknot connecting them. **a** The mapping that Kauffman and Magarshak proposed. **b** Another mapping in which the pseudoknot corresponds to the intersection between $s_1$ and $s_2$ (grey dot)

We can also discuss another research direction by analyzing different mappings from RNA secondary structures to tangles. For example, in the mapping we discussed in this paper, if there is a pseudoknot *p* connecting stems $s_1$ and $s_2$ then in the corresponding tangle there will be three edges, one for each of them. In this framework, the interaction between two stems is represented by an edge intersecting their corresponding edges. We could, instead, think of another mapping in which stems connected by a pseudoknot will have their corresponding edges that cross each other (Fig. 15).

We did not explore this alternative mapping, so we leave it as a future research direction.

Regarding the factorization algorithm, there are also some improvements that can be done with respect to the time complexity. Our methodology uses heuristics to obtain a non-minimal factorization and then refines it by using rewriting logic. This last step becomes prohibitive for large tangles, therefore a faster approach is necessary. During our research, we did not find an algorithm capable of such performances, but we have the hypothesis that the factorization problem for the Brauer Monoid could be solved in polynomial time.

Let’s discuss now some practical applications our methodology could be used for.

The factor representation we have discussed in this paper can be useful as an additional classification criterion for RNA secondary structures databases, in which a user could query RNAs that are generated only by a particular set of prime tangles, without the need of specifying the exact shape of the RNA molecule they are interested in. This could also lead to interesting applications in the context of sequence alignment, in which two sequences are compared not by the alignment of their nucleotides, but by their factor list.

As we discussed in “Background” section, the folding problem is the focus of a large amount of research. In recent years, Machine Learning techniques have been widely used in this context, in which a model is trained to predict the optimal secondary structure from a sequence of nucleotides [25]. We imagine that a machine learning model could be trained to predict the full factorization of the optimal secondary structure so that its shape would be easily computable or, alternatively, a model capable of predicting just a subset of this factorization, thus greatly reducing the search space for the optimal structure. We have not investigated this path, so we leave it as a future research direction.

## Conclusions

We have crossed the bridge that Kauffman and Magarshak have built between RNA secondary structures and the Brauer Monoid to pave the way for a novel prime tangle factorization for RNA secondary structures. Our results show that the presence of pseudoknots influences the type of factors the corresponding tangle has. Moreover, we proved that two interconnected sections of the RNA secondary structure will naturally share some factors. Since the exact interpretation of equivalent factorizaion is not clear, we expect further development in this direction. In any case, the proposed approach may reveal useful for reducing the search space for the optimal folding and for structure comparison and classification.

## Data Availability

Not applicable.

## Abbreviations

RNA: Ribonucleic acid
tRNA: Transfer RNA
